# On SFBC Schemes for Enabling Virtual Array Concept in Monostatic ISAC Scenarios †

**DOI:** 10.3390/s22031103

**Published:** 2022-02-01

**Authors:** Leonardo Leyva, Samuel Costa, Daniel Castanheira, Adão Silva, Atílio Gameiro

**Affiliations:** 1 Instituto de Telecomunicações (IT), Departamento de Eletrónica, Telecomunicações e Informática (DETI), University of Aveiro, 3810-193 Aveiro, Portugal; samuelcosta@ua.pt (S.C.); asilva@av.it.pt (A.S.); amg@ua.pt (A.G.); 2 Instituto de Telecomunicações (IT), 3810-193 Aveiro, Portugal; dcastanheira@av.it.pt

**Keywords:** MIMO, SFBC, OFDM, radar, virtual array, communication, ISAC

## Abstract

The integrated sensing and communication (ISAC) paradigm is being proposed for 6G as a new feature of the physical layer (PHY), for tackling dual-functional applications, i.e., demanding radio-sensing and communication functions, such as the Internet of Things (IoT) and autonomous driving systems. This work considers the integration of sensing and communications functionalities in a unique platform. To achieve this goal, the use of orthogonal space frequency block codes (SFBC) is proposed. SFBC code orthogonality enables both the separation of communications data streams at a user terminal and the estimation of target parameters. The SFBC enhances the communications link diversity without requiring channel state information knowledge at the transmitter and enable the virtual antenna array concept for enhancing the direction-finding resolution. The use of different SFBCs provides a tradeoff between achieved diversity and sensing resolution. For example, an Alamouti code, applicable for the case with two transmitting antennas, duplicates sensing resolution and achieves a diversity order of two while the use of a Tarokh code, applicable for a scenario with four transmitting antennas, provides a fourfold better resolution and diversity order of four. However, the code rate achieved with the Tarokh code is half of the one achieved with the Alamouti code. Furthermore, the unambiguous range is reduced since the bandwidth is divided to multiplex the different antenna signals. For its simplicity, good performance and reduced integration requirements, the method is promising for future ISAC systems.

## 1. Introduction

The hardware infrastructure saving, enhanced spectral/energy efficiency, and likely foreseen applications (e.g., Intelligent Transportation Systems (ITS), Internet of Things (IoT), and smart cities) have led integrated sensing and communication (ISAC) systems to be considered as a potential physical layer feature for 6G [1,2,3]. Furthermore, the technical similarities and commonly found technologies between communication and radar applications open the gate of a fully integrated system. For instance, the bandwidth of wireless systems is becoming the order of those used by radar (e.g., 5G is considering 400 MHz bandwidth). Moreover, technologies such as multiple-input multiple-output (MIMO), orthogonal frequency division multiplexing (OFDM) and its combination are common for both applications [4]. Proposals concerning stand-alone JCAS systems can be found in [2,5,6], while a networking JCAS paradigm is proposed by [3,7]. The work in [7] proposes the concept of a “perceptive mobile network”, which discusses the types of radio sensing that may be enabled by the network topology and the required network modification in terms of signaling. On the other hand, [3] presents an ultra-dense network (UDN) with radio-sensing capabilities, where the massive cooperation enables the cooperative multiterminal approach to deliver communication and sensing capabilities. These contributions highlight that OFDM and MIMO are enabler technologies in the development of future ISAC systems.

### 1.1. Related Work

For several decades, MIMO systems have been extensively deployed for commercial wireless communication systems. The use of multiple antennas and signal processing provides array, diversity and spatial multiplexing gains, which enhance the wireless system performance in terms of coverage, link reliability and capacity [8]. In addition to its popularity in wireless communication systems, MIMO has also been implemented for radar processing [9,10,11]. In this regard, MIMO radar has been shown to present benefits when compared to classical phased array radar such as higher resolution, and great flexibility regarding waveform design (i.e., from matching a desired beampattern to transmit orthogonal probing signals) [12,13]. MIMO radar with co-located antennas may enhance the direction identifiability (i.e., the maximum number of targets that can be uniquely identified in the direction domain) and the direction-finding resolution through the concept of virtual array [10,12]. Besides, [12] shows that the Cramér-Rao bound (CRB) of target parameter estimation is minimized when orthogonal probing signals are employed.

Orthogonal probing beams can be achieved through many waveforms, i.e., classical or communication-based radar. For instance, [2] proposed a three-stage ISAC method, where the initial stage employed a set of orthogonal frequency-modulated continuous waves (FMCW) for MIMO radar purposes. On the other hand, [14] used spectrally interleaved OFDM, which holds the orthogonality but allows for joint radar and communication applications, for a cooperative distributed target tracking method with application in vehicular environments. However, radar-like waveforms and spectrally interleaved OFDM lead to reduced diversity and spectral efficiency for communication purposes. Therefore, space–time block codes (STBC) or space–frequency block codes (SFBC) [15] stands as an option that brings diversity, while holding the probing signals orthogonality, which enables the virtual array concept for enhancing the direction-finding resolution for radar application. The contribution in [15] proposed a method to exploit the diversity provided by the STBC codes for secure radar imaging applications, where the waveform is designed to behave as a pseudo noise (PN) sequence. However, it lacks the estimation of the Doppler shift. More recently, [16] proposed an estimation method based on matched filters (MF) for a bistatic ISAC system. Here, the authors used pilots and correlation for a MIMO OFDM ISAC system. Although an interesting proposal, it has been shown that the correlation properties of OFDM, without any previous processing, reports several drawbacks, namely high computational complexity and range/Doppler ambiguities due to cyclic prefix (CP) and data dependency [5,17].

A simple processing algorithm for OFDM radar is proposed in [17], where the CP is first removed, and the data dependency is eliminated by a simple element-wise division between the baseband received signal and the transmitted symbols. This method was extended to estimate the Doppler shift, cope with a multi-path/multi-user scenarios and MIMO OFDM for direction finding [18]. Besides its simplicity, these methods—based on OFDM—provide the possibility of simultaneously conveying communication data and performing radio-sensing functionalities. For instance, [19] evaluated the performance of OFDM-based commercial communication waveforms for radio-sensing purposes, with a focus on Long-Term Evolution (LTE) and 5G New Radio (NR). However, the frequency misalignment in OFDM may be a serious drawback in real environments. The authors of [20] proposed a flexible and intuitive interference cancelation method for an ISAC paradigm to be performed when the radar estimation is no longer reliable. Besides, the delay-Doppler resolution provided by the OFDM waveform may be inadequate for use cases such as autonomous driving, where closely located (space and velocity) cars and pedestrians may compose the surrounding. In this regard [21], presented a high-resolution delay-Doppler estimation algorithm based on the subspace method. This contribution exploited the combination of the ISAC waveform backscattered from objects in the surrounding and pilots from the received communication signals to estimate target parameters.

### 1.2. Contribution

To our best knowledge, the use of STBC/SFBC to enable integrated sensing and communication has not yet been addressed in the literature. We consider that orthogonal SFBC stands as a technique that can be used to achieve the integration of radio sensing and communication on a common platform quickly and efficiently, reducing costs/hardware, and enhancing spectral efficiency. Besides, the junction of SFBC and co-located MIMO radar processing lead to enhanced direction-finding resolution of antenna arrays with reduced physical aperture size, which may be critical for a scenario with physical space constraints (e.g., autonomous driving).

This paper considers a monostatic MIMO-OFDM ISAC system, where the base station is equipped with a uniform linear array transmitting orthogonal probing signals. The orthogonality is achieved by coding the OFDM waveform with orthogonal SFBC in the space/frequency domain (e.g., Alamouti and Tarokh). It is assumed that the UEs have full knowledge of the channel frequency response and the SFBC decoding matrix, and in this way achieve full diversity. Meanwhile, the radar receiver is co-located with the transmitting antennas, enabling the virtual array concept. The radar signal processing exploits the knowledge of the orthogonal SFBC code and the transmitted symbols to estimate the direction, delay and Doppler through the received echo from targets. The proposed method exploits the full OFDM frame for delay/Doppler processing, which is different to the work in [16]. Besides, since the Doppler shift and data are first eliminated, the estimate is free of ambiguities and the complexity is considerably reduced compared to correlation approaches [5,17]. The performance of the estimation parameters is evaluated in terms of parameters resolution in a scenario considering several targets and for different SFBC codes. Besides, the SFBC schemes are compared with the interleaved approach [14,20] in terms of direction-finding resolution and BER.

To summarize, the major contributions of this paper are as follows:

- The integration of communication and radio-sensing functionalities in a single platform is achieved using SFBC. The orthogonality of SFBC enables the applicability of the virtual array concept and at the same time exploits the diversity of the communication channel.
- The virtual array concept leads to improved direction-finding resolution in the radio-sensing estimation of targets parameters.
- The proposed method is applicable for any orthogonal SFBC. The use of different SFBCs leads to a trade-off between communication rate and radio-sensing resolution.
- We compare the performance of SFBCs, which differs in the number of antennas and orthogonal code, in terms of radio sensing (i.e., direction-finding resolution and maximum unambiguous range) and communication metrics (i.e., rate and bit error rate).

The remainder of this paper is organized as follows: Section 2 generally describes the system model of the ISAC transmitter, the communication receiver structure and processing and the radar signal processing method for estimating the target parameters. Section 3 discusses several SFBC codes within the frame of the general structure described in Section 2. Section 4 is devoted to the performance results of the proposed monostatic ISAC scenario considering several targets and SFBC codes. A performance trade-off study between the results resulting from each SFBC code is given. Finally, Section 5 concludes the paper.

### 1.3. Notations

Boldface upper-case letters represent matrices and boldface lower-case letters denote a column vector. ||⋅|| and ${\left(\cdot \right)}^{H}$ represent Euclidean and conjugate transpose, respectively. $A_{:,n}$ stands as the column n of matrix **A**.

## 2. System Model

**Figure 1:**
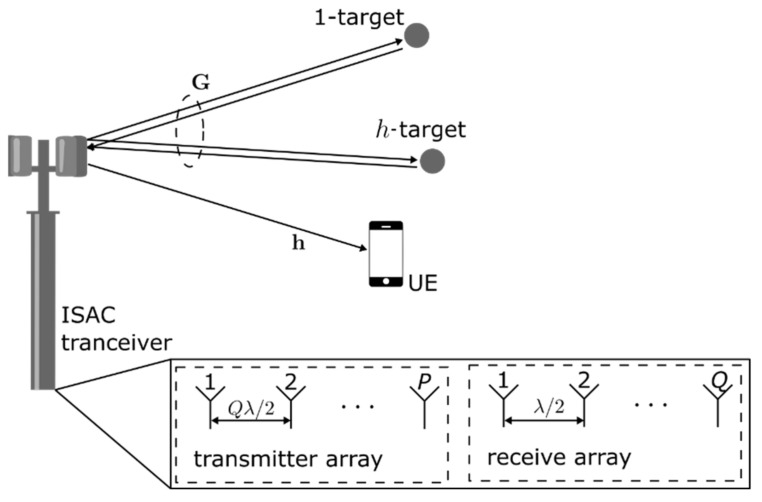
MIMO-OFDM monostatic ISAC scenario.

As illustrated in Figure 1, this work considers a monostatic MIMO-OFDM ISAC base station (BS), where the transmitter and receiver uniform linear arrays (ULA) are co-located.

The BS transmitted signal carries data information to a user equipment (UE) within its coverage area, which is equipped with a single antenna element. At the same time, the transmitted signal is scattered back from objects within the coverage area. The MIMO-OFDM ISAC BS receives the echoes and detects the presence of objects. The vector h and the matrix G represents the communication and radar channel, respectively. For communication processing, it is assumed that the channel is known at the communication terminal. For radar processing, the transmitted signal is assumed to be known at the radar receiver. In the following sections the ISAC transmitter, receiver and communication terminal are described.

### 2.1. ISAC Transmitter

The ISAC transmitter, the BS, is equipped with P transmitting antennas and Q receiving antennas. The transmitter antenna elements are spaced by a distance Qλ/2, where λ denotes the wavelength. The specific spacing of the transmitting antennas was selected such that it obeys the virtual array concept [12], which enhances direction-finding resolution. Besides, OFDM and orthogonal SFBC are considered, increasing the diversity order for the communications functionality and bringing the required orthogonality between the transmitted probing signals for MIMO radar processing.

**Figure 2:**
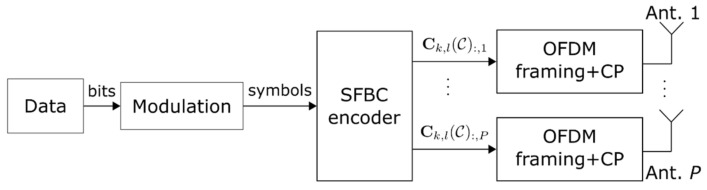
Block diagram of an ISAC transmitter.

Figure 2 presents the general block diagram of the ISAC transmitter. First the binary data stream is modulated and mapped from a digital constellation alphabet (e.g., QPSK, 16-QAM) into a sequence of symbols and divided into S data streams according to the SFBC scheme. Let $c_{s}(k,l)$ denote the s-th stream data symbol at subcarrier k∈{0,…,N−1} and OFDM symbol l∈{0,…,L−1}, then corresponding data vector for subcarrier k and symbol l is $c_{k,l}={\left[c_{1}\left(k,l\right),\ldots ,c_{S}\left(k,l\right)\right]}^{T}$. The SFBC scheme is defined by the matrix $C_{k,l}(C),\in {\mathbb{C}}^{N_{r}\times P}$, which encodes the data vector $c_{k,l}$, C being the selected code (e.g., Alamouti, Tarokh), and $N_{r}$ the number of frequency resources. The coding is then performed through the use of the respective coding matrix $C_{k,l}(C)$, where k={0,…,N−1} is the block subcarrier index and l={0,…,L−1} the OFDM symbol index. Then, for each antenna, the OFDM framing and cyclic prefix (CP) adding operations follow, as described in Figure 2.

### 2.2. Communication Terminal

This section describes in detail the signal model and the operations performed at the communication terminal for data detection.

#### 2.2.1. Signal Model

**Figure 3:**
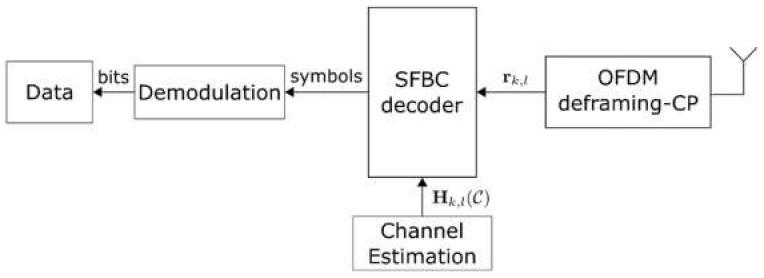
Block diagram of ISAC communication receiver.

Consider a communication device within the coverage area of the ISAC transmitter, which is equipped with a single antenna. We aim to recover the original data sent from the ISAC system. Figure 3 illustrates the block diagram of the communication terminal considered. Namely, the communication receiver includes the inverse operations of the transmitter. First the CP is removed then the OFDM signal is deframed, decoded and demodulated. At the output follows the data estimate.

Assuming that the coherence bandwidth of the channel is higher than the duration of $N_{r}$ adjacent sub-carriers, then the received signal model at the UE for the *k*-th frequency block and *l*-th OFDM symbol, after CP removal and FFT, can be expressed [22] as

(1) $$r_{k,l}=C_{k,l}\left(C\right)h_{k,l}+n_{k,l},$$

where, $h_{k,l}$ is the channel frequency response between the P transmitting antennas and the single receiving antenna and $n_{k,l}\sim CN(0,N_{0}I)$ denotes white Gaussian noise.

#### 2.2.2. Data Detection

The soft decision of subcarrier k and OFDM symbol l data vector is obtained by performing the operation,

(2) $${\tilde{c}}_{k,l}=H_{k,l}^{H}(C){\bar{r}}_{k,l},$$

where $H_{k,l}^{}(C)$ denotes the equivalent channel considering SFBC code C was used at the transmitter and ${\bar{r}}_{k,l}$ represents the equivalent received signal, whose structure depends on the SFBC scheme, as follows

(3) $${\bar{r}}_{k,l}=H_{k,l}(C)c_{k,l}+n_{k,l}.$$

Substituting (3) into (2), the next relation is obtained, where is easy to see that the ISI is completely removed.

(4) $${\tilde{c}}_{k,l}=||h_{k,l}|{|}^{2}c_{k,l}+H_{k,l}^{H}n,$$

The equality (3) follows from the equality $H_{k,l}^{H}(C)H_{k,l}(C)=||h_{k,l}|{|}^{2}I$. Section 3 provides detailed structures through examples.

### 2.3. RADAR Terminal

This section starts by describing the receiver main blocks and signal model, then follows the description of the channel model and finally the method to estimate the target parameters.

#### 2.3.1. Signal Model

**Figure 4:**
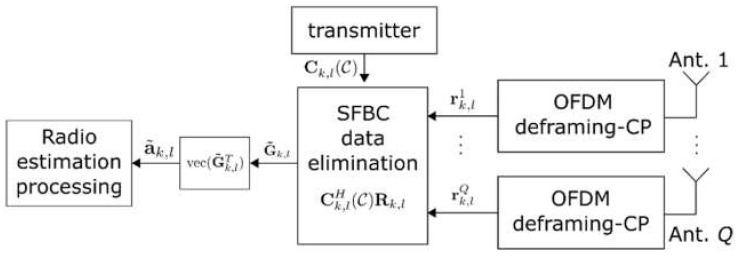
Co-located receiver schematic in MIMO-ISAC transceiver.

The radar receiver is co-located with the ISAC transmitter, i.e., monostatic radar topology. Figure 4 shows a schematic of the receiver in the MIMO-ISAC transceiver. The receiver ULA in Figure 4 is equipped with Q antenna elements spaced by a distance of λ/2, which enables the virtual array concept [12]. The objective of the radar receiver is to perform estimation of target parameters.

After CP removal and OFDM deframing, the received signal model at the *q*-th receiving antenna, for sub-carrier k and block l, is given [22] by

(5) $$r_{k,l}^{q}=C_{k,l}(C)g_{k,l}^{q}+n_{k,l}^{q},$$

where $g_{k,l}^{q}$ denotes the channel frequency response between the P transmitting antennas and the *q*-th antenna element of the receiver ULA and $n_{k,l}\sim CN(0,N_{0}I)$ denotes white Gaussian noise.

Let us define $G_{k,l}=[g_{k,l}^{1},\ldots ,g_{k,l}^{Q}]$, $R_{k,l}=[r_{k,l}^{1},\ldots ,r_{k,l}^{Q}]$ and $N_{k,l}=[n_{k,l}^{1},\ldots ,n_{k,l}^{Q}]$ as the concatenation of all receiving antennas signals, then from (5) follows

(6) $$R_{k,l}=C_{k,l}(C)G_{k,l}+N_{k,l},$$

#### 2.3.2. Channel Model

Considering that the transmitted signal is reflected by H targets the frequency domain response of the channel is described [2] by

(7) $$G_{k,l}=\sum_{h=1}^{H}a_{tx}\left(\theta_{h}\right)a_{rx}^{T}\left(\theta_{h}\right)e^{j2\pi T_{0}f_{D_{h}}l}e^{-j2\pi \Delta f\tau_{h}k}$$

where $f_{D}=2v/\lambda$ denotes the Doppler frequency, $\tau =2R/c_{0}$ the delay, ϕ=sin(θ) the electrical angle, $T_{0}$ the OFDM symbol duration, Δf the subcarrier spacing and $c_{0}$ the speed of light. $a_{tx}\left(\theta \right)$ and $a_{rx}\left(\theta \right)$ denote the transmitter and receiver array response vectors.

Let us define the vector $a_{k,l}=\mathrm{vec}(G_{k,l}^{T})$, where the operator vec(⋅) vectorizes the input matrix column by column, then from (7) follows that

(8) $$a_{k,l}=\sum_{h=1}^{H}a\left(\theta_{h}\right)e^{j2\pi T_{0}f_{D_{h}}l}e^{-j2\pi \Delta f\tau_{h}k}$$

where $a\left(\theta_{h}\right)=a_{rx}\left(\theta_{h}\right)⊗a_{tx}\left(\theta_{h}\right)$ is the array response vector of the virtual array formed by the transmitter and receiver ULA. As for the p th antenna element of the virtual array $a_{p}\left(\theta \right)=e^{-j\pi p\theta }$, the p th element of the vectorized channel is given by

(9) $$a_{k,l}\left(p\right)=\sum_{h=1}^{H}e^{-j\pi p\theta }e^{j2\pi T_{0}f_{D_{h}}l}e^{-j2\pi \Delta f\tau_{h}k}$$

#### 2.3.3. Target Parameter Estimation

The objective is now to recover the channel matrix $G_{k,l}$ by making use of the Hermitian property of the coding matrix $C_{k,l}^{H}(C)C_{k,l}(C)=I$, true for orthogonal SFBCs. The channel may be estimated as

(10) $$\begin{array}{c} {\tilde{G}}_{k,l}=C_{k,l}^{H}R_{k,l}\\ \;=G_{k,l}+C_{k,l}^{H}N_{k,l} \end{array}$$

where the last equality follows from $C_{k,l}^{H}C_{k,l}=I$. Therefore, from ${\tilde{G}}_{k,l}$ follows ${\tilde{a}}_{k,l}=\mathrm{vec}({\tilde{G}}_{k,l}^{T})$ where entry p is ${\tilde{a}}_{k,l}(p)$. The channel response $a_{k,l}\in {\mathbb{C}}^{PQ}$ is identical to the channel response of a system with one transmitting antenna and a uniform linear array with PQ receiving antennas with an inter-antenna distance of λ/2. This PQ-element array is the virtual antenna array [12], which is obtained with just P+Q physical antenna elements.

Accordingly to (9) the target parameters may be estimated by performing an DFT or IDFT along the three dimensions (k,l,p) of ${\tilde{a}}_{k,l}(p)$ [17]. The IDFT performed along the k dimension provides an estimative of the range, the DFT along the l dimension an estimative of the velocity and the IDFT along the p dimension provides an estimative of the electrical angle (target angle). For instance, for obtaining the delay-Doppler radar image, a well-known tool for resolving sinusoids, based on DFT and IDFT, known as the two-dimensional complex periodogram [17] can be used,

(11) $$\mathrm{Per}(n,m)=\frac{1}{NL}{\left|\sum_{n=0}^{N-1}\left(\sum_{l=0}^{L-1}{\tilde{a}}_{k,l}(p)e^{-j2\pi \frac{lm}{L}}\right)e^{j2\pi \frac{kn}{N}}\right|}^{2}$$

where the (n,m) pair stands as a bin of the periodogram, with n∈{0,…,N−1} and m∈{0,…,L−1}. A sinusoid in ${\tilde{a}}_{k,l}(p)$ results in a peak in $\mathrm{Per}(\hat{n},\hat{m})$, which corresponds to the range-velocity bin of the target. Similar processing to (11) yields the angle–velocity or range–angle radar images [17].

## 3. SFBC Practical Examples

In the previous section the proposed method was described considering a generic SFBC. In the following, the approach is detailed for specific SFBCs, namely Alamouti and Tarokh codes. As the approach is quite similar for both SFBCs, only the key aspects regarding each code are presented.

### 3.1. SFBC Alamouti

Following the Alamouti approach, after the modulation stage, the symbols are separated in two data streams $c_{1}$ and $c_{2}$. The data streams are then coded into a two frequency-slot block described by the presented matrix $C_{k,l}$

(12) $$C_{k,l}=\left[\begin{array}{cc} c_{1}(k,l) & c_{2}(k,l)\\ -c_{2}^{*}(k,l) & c_{1}^{*}(k,l) \end{array}\right]$$

where column 1 (2) of matrix $C_{k,l}$ corresponds to the signal to be transmitted on antenna 1 (2), and decoded using the Hermitian of the equivalent channel matrix defined by

(13) $$H_{k,l}=\left[\begin{array}{cc} h_{k,l}(1) & h_{k,l}(2)\\ h_{k,l}^{*}(2) & -h_{k,l}^{*}(1) \end{array}\right]$$

### 3.2. SFBC Tarokh

In the Tarokh approach after the modulation stage, the symbols are separated in four data streams $c_{1}$, $c_{2}$, $c_{3}$ and $c_{4}$. These data streams are then coded in an eight frequency-slot block described by the presented matrix $C_{k,l}$. As an example, let us consider a Tarokh code with P=4 antennas and $N_{r}=8$ frequency resources, with a code rate of 1/2.

(14) $$C_{k,l}=\left[\begin{array}{cccc} c_{1}\left(k,l\right) & c_{2}\left(k,l\right) & c_{3}\left(k,l\right) & c_{4}\left(k,l\right)\\ -c_{2}\left(k,l\right) & c_{1}\left(k,l\right) & -c_{4}\left(k,l\right) & c_{3}\left(k,l\right)\\ -c_{3}\left(k,l\right) & c_{4}\left(k,l\right) & c_{1}\left(k,l\right) & -c_{2}\left(k,l\right)\\ -c_{4}\left(k,l\right) & -c_{3}\left(k,l\right) & c_{2}\left(k,l\right) & c_{1}\left(k,l\right)\\ {c_{1}}^{*}\left(k,l\right) & {c_{2}}^{*}\left(k,l\right) & {c_{3}}^{*}\left(k,l\right) & {c_{4}}^{*}\left(k,l\right)\\ -{c_{2}}^{*}\left(k,l\right) & {c_{1}}^{*}\left(k,l\right) & -{c_{4}}^{*}\left(k,l\right) & {c_{3}}^{*}\left(k,l\right)\\ -{c_{3}}^{*}\left(k,l\right) & {c_{4}}^{*}\left(k,l\right) & {c_{1}}^{*}\left(k,l\right) & -{c_{2}}^{*}\left(k,l\right)\\ -{c_{4}}^{*}\left(k,l\right) & -{c_{3}}^{*}\left(k,l\right) & {c_{2}}^{*}\left(k,l\right) & {c_{1}}^{*}\left(k,l\right) \end{array}\right]$$

where columns 1 to 4 of matrix $C_{k,l}$ correspond to the signal to be transmitted on antennas 1 to 4, respectively. The decoding operation for the Tarokh code is similar to the one performed for the Alamouti code, but now using the Hermitian of the equivalent channel matrix given by

(15) $$H_{k,l}=\left[\begin{array}{cccc} h_{k,l}\left(1\right) & h_{k,l}\left(2\right) & h_{k,l}\left(3\right) & h_{k,l}\left(4\right)\\ -h_{k,l}\left(1\right) & h_{k,l}\left(2\right) & -h_{k,l}\left(3\right) & h_{k,l}\left(4\right)\\ -h_{k,l}\left(1\right) & h_{k,l}\left(2\right) & h_{k,l}\left(3\right) & -h_{k,l}\left(4\right)\\ -h_{k,l}\left(1\right) & -h_{k,l}\left(2\right) & h_{k,l}\left(3\right) & h_{k,l}\left(4\right)\\ h_{k,l}{\left(1\right)}^{*} & h_{k,l}{\left(2\right)}^{*} & h_{k,l}{\left(3\right)}^{*} & h_{k,l}{\left(4\right)}^{*}\\ -h_{k,l}{\left(1\right)}^{*} & h_{k,l}{\left(2\right)}^{*} & -h_{k,l}{\left(3\right)}^{*} & h_{k,l}{\left(4\right)}^{*}\\ -h_{k,l}{\left(1\right)}^{*} & h_{k,l}{\left(2\right)}^{*} & h_{k,l}{\left(3\right)}^{*} & -h_{k,l}{\left(4\right)}^{*}\\ -h_{k,l}{\left(1\right)}^{*} & -h_{k,l}{\left(2\right)}^{*} & h_{k,l}{\left(3\right)}^{*} & h_{k,l}{\left(4\right)}^{*} \end{array}\right]$$

## 4. System Performance

In this section, the performance of the proposed ISAC system is presented and evaluated for all the previously mentioned approaches. Results are presented both for the communication and radar functionalities, first considering the use of Alamouti and then Tarokh SFBCs. Namely for the communication the metric under consideration is the bit-error-rate (BER) and for the radar the corresponding radar images are presented. Finally, the results obtained for the two codes are compared and final conclusions drawn.

### 4.1. ISAC Specifications and Scenarios Description

As pointed out, the channel can be modeled as a sparse mmWave channel made-up of *H* paths, as in (7), from which it is desired to estimate the parameters of the paths, i.e., range, velocity and angle-of-arrival (AoA). Therefore, to validate SFBC for radio-sensing applications, first the two-dimensional range-velocity/AoA-velocity radar imaging obtained for several targets are presented. Then, analysis of the resolution of the AoA is given for several SFBC and receiving antennas. Finally, the BER performance for several SFBCs and modulation schemes is presented.

The MIMO OFDM ISAC system operates at $f_{c}$ = 24 GHz ISM mmWave band, which has been used for research purposes as it is deregulated. The range–velocity bin is estimated through a complex periodogram [17] over the OFDM frame. Therefore, the OFDM parameters are chosen to fulfil a set of design criteria as, maximum unambiguous range $r_{unamb}$, maximum unambiguous velocity $v_{unamb}$, range resolution Δr and velocity resolution Δv. The design criteria and OFDM parameters are summarized in Table 1.

### 4.2. Results for Alamouti and Tarokh

First, for validating the SFBC for radio-sensing applications, we consider an ISAC scenario with two transmitting antennas (Alamouti SFBC, *P* = 2), eight receiving antennas (*Q* = 8), and eight targets (*H* = 8), from which the range, velocity and AoA parameters are summarized in Table 2.

**Figure 5:**
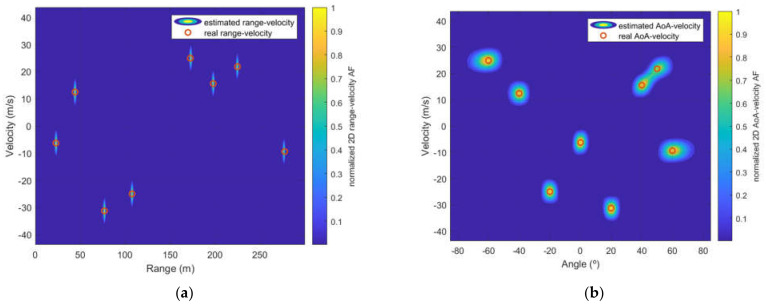
Radar imaging obtained for Alamouti SFBC and eight receiver antennas, (**a**) normalized 2D range-velocity AF obtained for targets in Table 2, (**b**) two-dimensional AoA-velocity AF obtained for targets in Table 2.

Figure 5 illustrates the normalized range–velocity and AoA-velocity AF obtained for targets in Table 2. At a glance, it can be seen that signals resulting from Alamouti SFBC can be exploited for radio-sensing applications.

**Figure 6:**
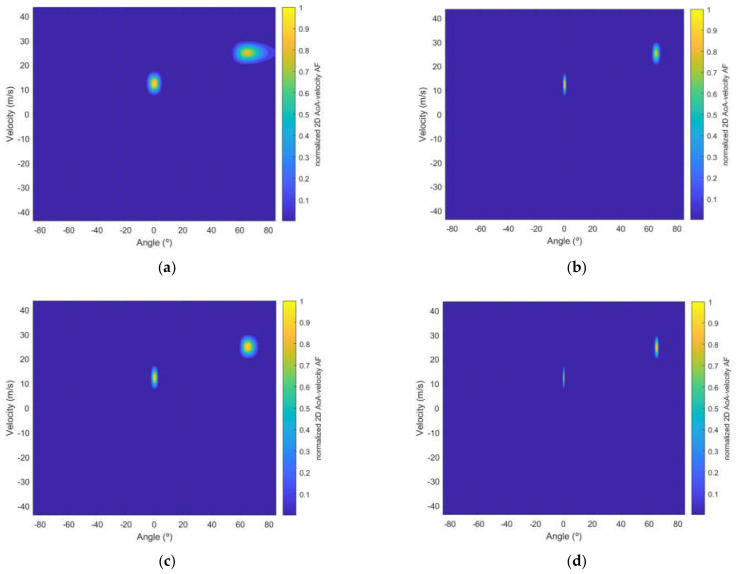
Normalized 2D AoA-velocity AF obtained for two targets with a (velocity, AoA) equal to (12.4914 m/s, 0°) and (24.9827 m/s, 65°) for scenarios: (**a**) Alamouti SFBC and receiver ULA with 8 antenna elements; (**b**) Alamouti SFBC and receiver ULA with 32 antenna elements; (**c**) Tarokh SFBC and receiver ULA with 8 antenna elements and (**d**) Tarokh SFBC and receiver ULA with 32 antenna elements.

For the analysis of AoA estimation, Alamouti and Tarokh SFBC and different ULA configurations of the receptor are considered. Figure 6 shows a scenario with two targets with a velocity and AoA equal to (12.4914 m/s, 0°) and (24.9827 m/s, 65°), respectively. Results are obtained for the Alamouti and Tarokh codes considering 2 and 4 transmitting antennas, respectively. For the receiver both an ULA with 8 and 32 antennas are considered.

**Figure 7:**
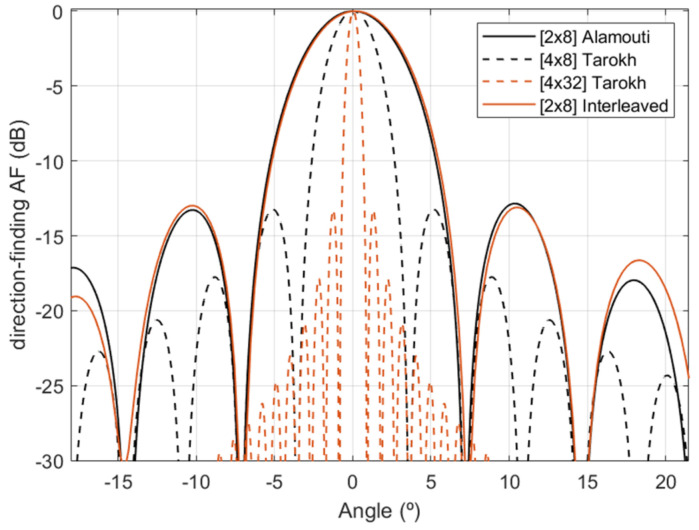
Direction-finding resolution comparison between SFBCs schemes and interleaved approach for a target located at 0°.

From Figure 6, it can be noticed that when the angle approaches 90 degrees (e.g., target at 65°), the resolution attained degrades when compared to the target located at 0°, this effect is more evident in Figure 5a, where the resolution is distorted from 65°. Additionally, for both Alamouti and Tarokh scenarios, the direction-finding resolution improves when the number of receiver antennas increases. A comparison of the direction-finding resolution between SFBC and the interleaved waveform approach in [14,20] is presented in Figure 7.

**Figure 8:**
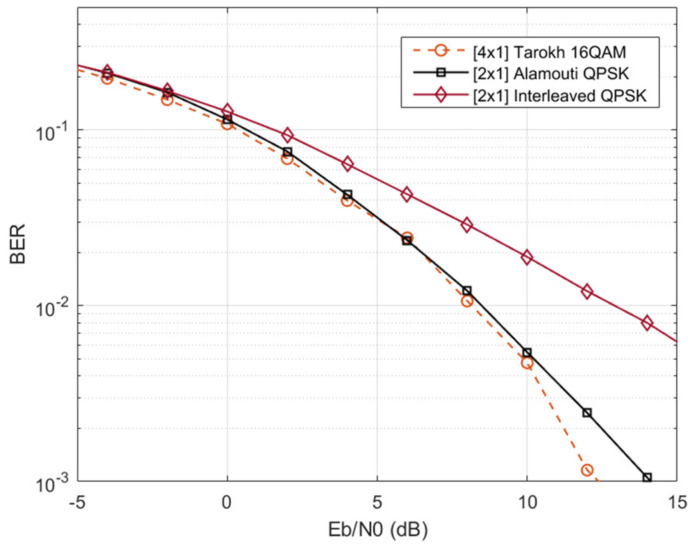
BER performance comparison.

From Figure 7, it can be noticed that Alamouti SFBC with eight physical receiving antennas and the interleaved approach presents a similar direction-finding resolution. Additionally, they show less resolution than Tarokh SFBC with eight physical receiving antennas. This is a direct consequence of the *PQ* virtual array configuration. Although Tarokh SFBC presents better direction-finding resolution, it requires eight frequency resources; this is *N_r_* = 8, and therefore the unambiguous range reduces to 1.64 μs, which is less than the maximum unambiguous range of 2 μs chosen by design. This implies that by implementing Tarokh, targets located between 246 and 300 m cannot be estimated. Finally, Figure 8 illustrates a BER comparison between the SFBCs schemes and the interleaved approach [14,20].

Figure 8 shows that as in legacy wireless communication systems, the use of orthogonal SFBCs bring an improvement in the BER, i.e., the 2 × 1 Alamouti QPSK reports better BER than the 2 × 1 interleaved QPSK scheme. Additionally, the MISO-OFDM Tarokh 16-QAM and Alamouti QPSK report a similar BER. However, Alamouti outperforms Tarokh regarding efficiency, since it has a rate of one, while Tarokh has a rate of a half, i.e., Tarokh use twice the bandwidth as Alamouti for transmitting the same data. SFBC is therefore a promising candidate for the integration of radar and communications. The structure of the SFBC signals allows the formation of virtual arrays, achieving the same performance of interleaved orthogonal signaling, while enabling diversity in the communication domain, a feature that does not occur with orthogonal inter-leaving.

## 5. Conclusions

In this work, we have proposed the use of SFBC for the integration of radar and communication functionalities. The SFBC orthogonality properties provide the orthogonality necessary for MIMO radar to multiplex the different antenna signals and at the same time enhance the diversity order achieved by the communication functionality. These allow the radar resolution to be improved significantly by resorting to the virtual array concept, and the added diversity significantly reduces the resulting BER. The use of different SFBCs allows a tradeoff to be made between the achieved communication and radar performance. For example, the Alamouti code, applicable for the case with two transmitting antennas duplicates the radar spatial resolution and achieves diversity order 2. On the other hand, the Tarokh code, applicable for the case with four transmitting antennas, improves the radar spatial resolution by a factor of four, together with the diversity, but the code rate is reduced by half. Additionally, we compared the performance between SFBC schemes and the interleaved approach in terms of direction-finding resolution and BER. It was shown that for the same virtual array size, SFBC and interleaved report similar resolution. However, for the communication with interleaved, we lose the diversity benefit, and we have worse BER. Therefore, for the objectives of joint radar and communication, the use of SFBC is the preferred solution. Due to its low complexity, good performance, and reduced integration requirements, the method is promising for future ISAC systems.

## Figures and Tables

**Table 1:** OFDM and design criteria parameters for ISAC system.

Parameters	Value
Bandwith B	70.06 MHz
Maximum unambiguous range $r_{unamb}$	300 m
Maximum unambiguous velocity $v_{unamb}$	43.75 m/s
Range resolution Δr	3.84 m
Velocity resolution Δv	6.46 m/s
Number subcarriers $N_{c}$	1024
Subcarrier spacing Δf	76.230 kHz
Cyclic prefix duration $\tau_{CP}$	2 μs
Total OFDM symbol duration $T_{0}$	15.11 μs
Number of OFDM symbols L	64

**Table 2:** Target parameters.

Target	Range (m)	Velocity (m/s)	AoA (°)
Target 1	172	25	−60
Target 2	44	12	−40
Target 3	107	−25	−20
Target 4	23	−6	0
Target 5	77	−31	20
Target 6	198	16	40
Target 7	225	22	50
Target 8	277	−9	60

## Data Availability

Not applicable
